# Model-Free RNA Sequence and Structure Alignment Informed by SHAPE Probing Reveals a Conserved Alternate Secondary Structure for 16S rRNA

**DOI:** 10.1371/journal.pcbi.1004126

**Published:** 2015-05-20

**Authors:** Christopher A. Lavender, Ronny Lorenz, Ge Zhang, Rita Tamayo, Ivo L. Hofacker, Kevin M. Weeks

**Affiliations:** 1 Department of Chemistry, University of North Carolina, Chapel Hill, Chapel Hill, North Carolina, United States of America; 2 Institute for Theoretical Chemistry, University of Vienna, Vienna, Austria; 3 Bioinformatics Group, Department of Computer Science, University of Leipzig, Leipzig, Germany; 4 Department of Microbiology and Immunology, University of North Carolina, Chapel Hill, Chapel Hill, North Carolina, United States of America; 5 Bioinformatics and Computational Biology, University of Vienna, Vienna, Austria; University of Texas at Austin, UNITED STATES

## Abstract

Discovery and characterization of functional RNA structures remains challenging due to deficiencies in *de novo* secondary structure modeling. Here we describe a dynamic programming approach for model-free sequence comparison that incorporates high-throughput chemical probing data. Based on SHAPE probing data alone, ribosomal RNAs (rRNAs) from three diverse organisms – the eubacteria *E*. *coli* and *C*. *difficile* and the archeon *H*. *volcanii* – could be aligned with accuracies comparable to alignments based on actual sequence identity. When both base sequence identity and chemical probing reactivities were considered together, accuracies improved further. Derived sequence alignments and chemical probing data from protein-free RNAs were then used as pseudo-free energy constraints to model consensus secondary structures for the 16S and 23S rRNAs. There are critical differences between these experimentally-informed models and currently accepted models, including in the functionally important neck and decoding regions of the 16S rRNA. We infer that the 16S rRNA has evolved to undergo large-scale changes in base pairing as part of ribosome function. As high-quality RNA probing data become widely available, structurally-informed sequence alignment will become broadly useful for *de novo* motif and function discovery.

## Author Summary

Despite the clear functional importance of structure in RNA molecules, it remains very difficult to correctly identify and annotate similar RNA structures because their sequences are often poorly conserved even for RNAs that form very similar higher-order structures. A solution is to use a metric that identifies structural motifs but this, too, is difficult because RNA structure modeling based on sequence alone is generally not very accurate. In this work, we use SHAPE chemical probing to obtain model-free information about RNA structure and then exploit this information to align RNAs by sequence. We show for ribosomal RNAs that sequence alignments based on SHAPE experimental information alone are as accurate as those that actually use sequence information. In addition, we identify regions in the 16S ribosomal RNA that form conserved secondary structures that are different from currently accepted models. These differences, rather than being errors, reveal sites of conformational flexibility that may underlie mechanistic functions in ribosome assembly and translation regulation. We anticipate that structure-informed sequence alignment and structure modeling will become broadly useful tools in RNA function analysis.

## Introduction

RNA is a central participant in gene expression and regulation [1]. However, for a vast majority of RNA transcripts, the positions and roles of higher-order structure are unknown. Sequence comparison approaches can be powerful tools in the discovery and annotation of functional RNA motifs. In related functional RNAs, critical structural elements are conserved despite changes in primary sequence. As RNA structure appears to be more conserved than primary sequence [2, 3], functional RNA discovery and transcriptome annotation can be improved by taking into account RNA structure. Currently, structure-guided RNA sequence comparison approaches perform poorly and are limited by the pervasive difficulty of predicting RNA structures from sequence alone [4–6]. Moreover, optimization and benchmarking of current structure prediction approaches are confined to known RNA structure motifs, themselves limited to structures that are amenable to high-resolution structure characterization or comparative sequence analysis. RNA structure modeling is thus biased by a small number of well-characterized elements.

RNA comparison and alignment that considers some model-free metric of underlying structure is an attractive alternative to comparisons that use *ab initio* or concurrent structure prediction. RNA chemical probing is structurally robust and is not limited by the current, relatively poor, understanding of RNA structure. The SHAPE structure probing approach [7, 8] interrogates virtually all nucleotides of any RNA target. The adaptation of RNA chemical probing approaches to readout by massively parallel sequencing allows for high-throughput analysis and is rapidly advancing toward transcriptome-scale assays [9, 10].

In this work, we introduce and evaluate a sequence comparison approach that considers chemical probing data. We find that SHAPE-directed alignment, performed entirely independently of base identity information, generates sequence alignments with accuracies comparable to traditional nucleobase identity-directed methods. Approaches that consider both SHAPE reactivities and base identity improve accuracy relative to approaches considering base identity or chemical probing data alone. Chemical probing data were compared using a simple, general pair-wise scoring function that is broadly applicable to diverse sequence comparison methods and should significantly facilitate discovery of novel structurally conserved functional RNA motifs in large RNAs. SHAPE-directed alignments were then used to predict RNA secondary structures conserved among diverse ribosomal RNAs. Novel base pairing patterns were identified in 16S rRNA, suggestive of new RNA-based features of ribosome function.

## Results

### Selection and characterization of test-case RNA molecules

Ribosomal RNA was used for development and evaluation of SHAPE-dependent RNA structure alignment. Ribosomal RNA has been extensively characterized: Currently 83% of RNA nucleotides in high-resolution structures in the RCSB Data Bank belong to ribosomal RNAs. Thousands of ribosomal RNA sequences have been curated and aligned, with secondary and tertiary structures predicted based on covariation analysis [11]. Moreover, the diverse secondary and tertiary structure elements found in ribosomal RNA form the basis for much of our structural knowledge of RNA. RNA structure analysis and modeling methods based on analysis of ribosomal sequences have proven robust when extended to other RNAs [4, 12, 13].

Ribosomal RNA samples were obtained from three cultured organisms, eubacteria *Escherichia coli* and *Clostridium difficile* and archaea *Haloferax volcanii*. Differences among these organisms are reflected in the distinct culturing and cell lysis conditions required for each (see Methods). The ribosomal RNAs from these organisms are highly diverse; compared to *E*. *coli*, *C*. *difficile* and *H*. *volcanii* have percent nucleotide identities of only 72.6% and 59.7%, respectively. The three ribosomal samples were analyzed by SHAPE probing in which local nucleotide structural flexibility at a given position is determined by the extent of modification by a chemical probe [7]. Quantitative, nucleotide-resolution SHAPE reactivity values were determined using our recently described mutational profiling (MaP) approach, in which chemical modifications are recorded as mutation rates in the cDNA products generated during reverse transcription of chemically-modified RNAs [10]. Chemical modification-induced mutations are quantified with nucleotide resolution using massively parallel sequencing. SHAPE-MaP data for *E*. *coli* ribosomal RNA were reported previously [10]; SHAPE-MaP data for *C*. *difficile* and *H*. *volcanii* were newly obtained in this work.

### SHAPE reactivity data for related RNA nucleotides

**Fig 1:**
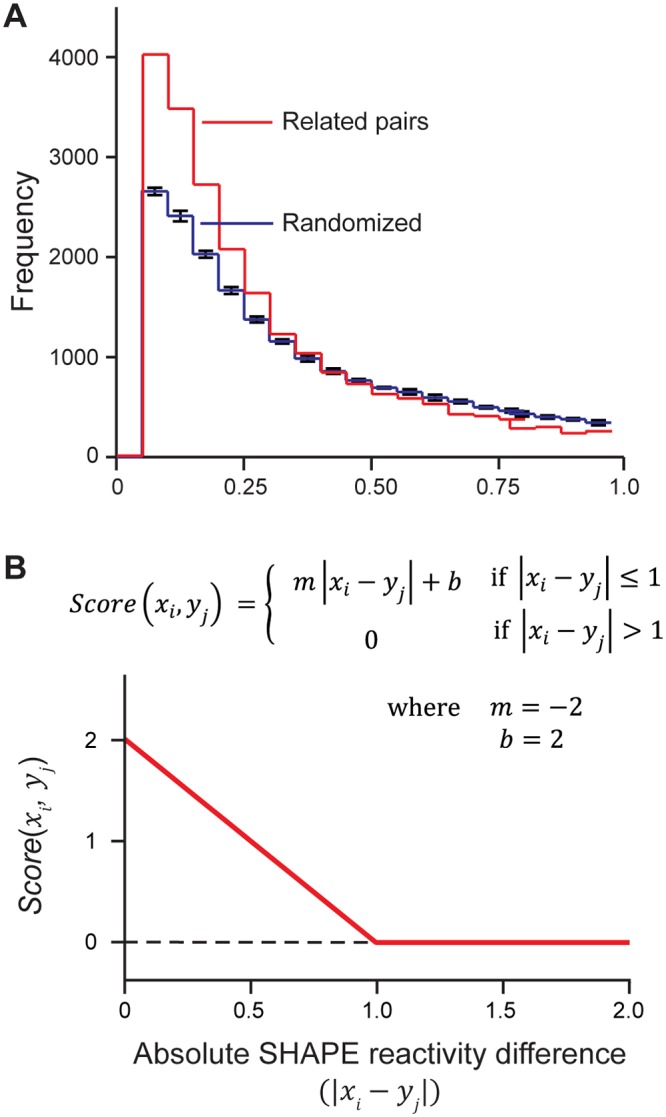
SHAPE-based scoring function for structurally-informed RNA sequence alignment. (**A**) Histogram of the absolute differences in SHAPE reactivities for paired nucleotides in accepted alignments. Differences between related pairs are shown in red, and differences between randomized pairs are blue. Pairs were randomized in eight individual trials; average values are shown with standard deviations given as error bars. (**B**) Scoring function used to compare SHAPE values at positions *i* and *j* in sequences *x* and *y*, respectively.

We first characterized the relationship between SHAPE reactivities for related RNA nucleotides in accepted sequence alignments. Related nucleotides for the ribosomal RNAs studied here were defined using annotated sequence comparisons from the Comparative RNA Web Site and Project (CRW) [11]. Related nucleotides were taken as nucleotide pairs in CRW alignments. For the 16S and 23S ribosomal RNAs, 24,467 nucleotide pairs were considered; for each nucleotide pair, we calculated absolute differences in SHAPE reactivities (Fig 1A, in red). The distribution of SHAPE reactivity differences in related RNA nucleotides followed an exponential decay. When SHAPE data were randomly resorted, differences in SHAPE reactivities between two related nucleotides were, on average, smaller than the differences between unrelated nucleotides (Fig 1B, in blue). The distribution describing related nucleotides is significantly different from the distribution describing randomly resorted nucleotide pairs (*p*-value< 10^-6^, Student's t-test).

### SHAPE-based scoring function and alignment

Global SHAPE-dependent sequence comparisons were performed using a pair-wise dynamic programming algorithm (see Methods) [14]. The algorithm uses recursion to align two sequences based on a pair-wise scoring function between individual nucleotides. The algorithm also incorporates penalties based on gap openings and gap extensions, where gaps are unaligned regions of sequence. We implemented a SHAPE comparison scoring function where small differences in SHAPE reactivities were given high (favorable) scores. Alignments were ultimately scored as the sum of individual SHAPE comparison scores and gap penalties over the entire alignment. Pair-wise SHAPE comparisons were scored by a linear function (Fig 1B), and scoring function parameters and gap opening and extension penalties were optimized by an exhaustive parameter search. Parameters were selected on the basis of sensitivity of alignments (fraction of aligned nucleotides shared with the accepted alignment) generated for 16S and 23S ribosomal RNAs relative to CRW pairwise alignments [11].

### Quality of SHAPE-based alignments

**Fig 2:**
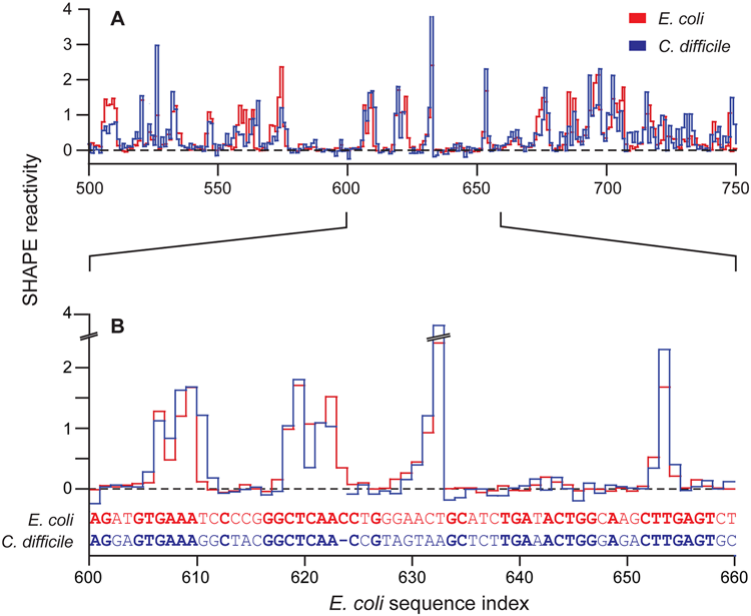
Representative global sequence alignment between *E*. *coli* and *C*. *difficile* 16S ribosomal RNAs using model-free SHAPE reactivities as the only constraint. Alignment is shown as a function of *E*. *coli* sequence numbering. (**A**) Alignment of SHAPE reactivities across a 250-nucleotide window. (**B**) A 60-nucleotide subsection of this alignment, including primary sequences. Areas of sequence identity are emphasized in bold.

SHAPE-based alignments were performed for all 16S and 23S ribosomal RNA pairs (Fig 2). The accuracies of the SHAPE-only alignments were compared to global sequence alignments obtained with the Needle algorithm [14, 15]. Sequence alignments based only on SHAPE data (without using any sequence information) were comparable in quality to Needle-based sequence alignments (Table 1). For example, for 16S rRNA, SHAPE-based alignments had sensitivities of 83% and 71% for alignments of *E*. *coli* to *C*. *difficile* and to *H*. *volcanii*, respectively; whereas, conventional sequence identity-based alignments using the Needle algorithm had sensitivities of 84% and 72%, respectively. For 23S rRNA, SHAPE-based alignments performed well but not quite at the level of sequence-based alignment (Table 1).

**Table 1:** Sensitivities of pairwise SHAPE-dependent sequence alignments relative to accepted alignments.

Sequence 1	Sequence 2	Sensitivity relative to CRW alignments (%)			
		Nucleobase identity	SHAPE-only	Combined SHAPE and nucleobase identity	
				Pairwise	MSA
*E*. *coli* 16S	*C*. *difficile* 16S	84	83	94	94
	*H*. *volcanii* 16S	72	71	89	92
*E*. *coli* 23S	*C*. *difficile* 23S	83	73	94	95
	*H*. *volcanii* 23S	58	41	76	79

SHAPE-only sequence alignments did not use sequence information. Accepted alignments are from the CRW [11]. Nucleobase identity-based alignment used the Needle algorithm [14] on the EMBOSS server [15] with default parameters. For predictions incorporating both SHAPE reactivities and nucleobase identity, sensitivities are given for both pairwise comparisons and for multiple sequence alignments (MSA) generated by T-Coffee [16].

### Incorporating a base-identity match score into SHAPE-based alignments

An additional scoring term considering base identity was then included in the alignment algorithm, allowing sequence comparison based on both base identity and SHAPE structure reactivity values. In a pairwise comparison, if two nucleotides had the same base identity, the pair was scored as a match, and a match bonus was included in the scoring function. Otherwise, a mismatch penalty was included. These scores were added to the values generated by the SHAPE comparison function. The score terms associated with both matches and mismatches were optimized by a parameter search. Gap opening and gap extension penalties were also re-optimized given this new scoring system. Alignments considering both sequence identity and SHAPE data showed significant improvements relative to alignments considering SHAPE structure data or base identity alone (Table 1). For 16S rRNA, alignments of *E*. *coli* to *C*. *difficile* and to *H*. *volcanii* had sensitivities of 94% and 92%, respectively, when both base identity and SHAPE data are considered. The *E*. *coli* 23S rRNA alignments to *C*. *difficile* and to *H*. *volcanii* had sensitivities of 89% and 84%, respectively.

We note that we performed benchmarking and parameter optimization using the same RNAs. This is because there are very few large RNA molecules with accepted sequence alignments, especially for culturable organisms. In independent work, we have applied SHAPE-based alignment, using the parameters defined using our three-species ribosomal RNA training data, to align single-stranded HIV-related viral RNA genomes. The viral RNAs constitute a fully independent test set. Based on nucleobase identity and SHAPE reactivities, HIV-1 strain NL4-3 (9,173 nts) and SIVcpz strain MB897 (9,167 nts; 77% sequence identity) aligned with a sensitivity of 97% relative to extensively manually curated and hand-annotated alignments (see following companion article in this issue).

Pairwise SHAPE-based alignments were also used to generate multiple sequence alignments [16]. Alignment quality did not change significantly with the multiple sequence alignment (Table 1). Alignment quality may increase in future applications with larger numbers of diverse sequences. Lack of significant change also likely reflects that the quality of the pairwise alignments was already high, leaving relatively little room for improvement.

### Secondary structure modeling with SHAPE-directed alignments

Both sequence comparisons and SHAPE data have been successfully used to direct RNA secondary structure modeling [17]. Given that SHAPE-based alignments effectively combine both of these classes of information, we examined the usefulness of using SHAPE-based alignments to model secondary structures. Sequence comparison-based secondary structure predictions are highly dependent on alignment quality [18, 19]. Therefore, success in secondary structure prediction would offer further support for model-free SHAPE-based alignment.

The SHAPE-defined sequence alignments were incorporated as arguments for secondary structure modeling using the RNAalifold algorithm in the Vienna RNA package [20–22]. RNAalifold uses a pseudo-free energy potential to bias predictions based on covariation information. In this scheme, base pairs supported by covariation are given a free-energy bonus. For this work, RNAalifold was updated to accept SHAPE reactivities as an additional pseudo-free energy term [4, 23]. Free energy calculations with RNAalifold were therefore the sum of three terms corresponding to thermodynamic parameters, sequence covariation, and SHAPE reactivity. A consensus structure from the sequence alignment was obtained from a partition function calculation as base pairs with pairing probabilities greater than 95%. Overall, ~77% of base pairs were identified in this first step (reported as sensitivity values in Table 2, column 4). Consensus base pairs identified in the first step were in turn used to constrain modeling of individual structures for each RNA in a given alignment, allowing additional base pairs to be identified. Finally, structure prediction was performed by free-energy minimization that included a pseudo-free energy term based on SHAPE reactivities.

**Table 2:** Sensitivities (sens) and positive predictive values (ppv) for secondary structure models as a function of included information.

RNA	(1) Individual models based on sequence alone		(2) Consensus models including sequence-only alignments		(3) Individual models based on SHAPE reactivities alone		(4) SHAPE-directed alignment consensus models, pairs with >95% pairing probability		(5) Individual models constrained by consensus pairs from SHAPE-directed alignment		(6) Individual models constrained by consensus pairs, structures with incompatible SHAPE data omitted	
	sens	ppv	sens	ppv	sens	ppv	sens	ppv	sens	ppv	sens	ppv
*E*. *coli* 16S	60.0	52.8	66.5	68.8	90.0	84.0	76.8	91.7	93.8	89.0	96.8	89.0
*C*. *difficile* 16S	58.7	51.8	70.9	70.1	89.7	83.8	79.6	90.4	92.7	86.6	95.6	86.6
*H*. *volcanii* 16S	78.1	68.3	59.3	69.1	89.3	81.5	74.9	91.2	90.2	85.3	92.2	85.3
*E*. *coli* 23S	68.7	59.8	60.0	64.1	85.6	78.3	79.0	89.1	90.7	83.1		

All values are given as percentages. Consensus structures were generated by RNAalifold [21] using SHAPE-directed sequence alignments (column pair 4). These structures were then used to constrain individual structure predictions (column pairs 5 and 6). Models were compared against three control predictions: individual predictions based on sequence alone (column 1), consensus predictions based on sequence-only alignment (column 2), and individual predictions made using only SHAPE reactivities and no sequence alignment information (column 3). Covariation structures are not available for *C*. *difficile* or *H*. *volcanii* 23S rRNAs.

**Fig 3:**
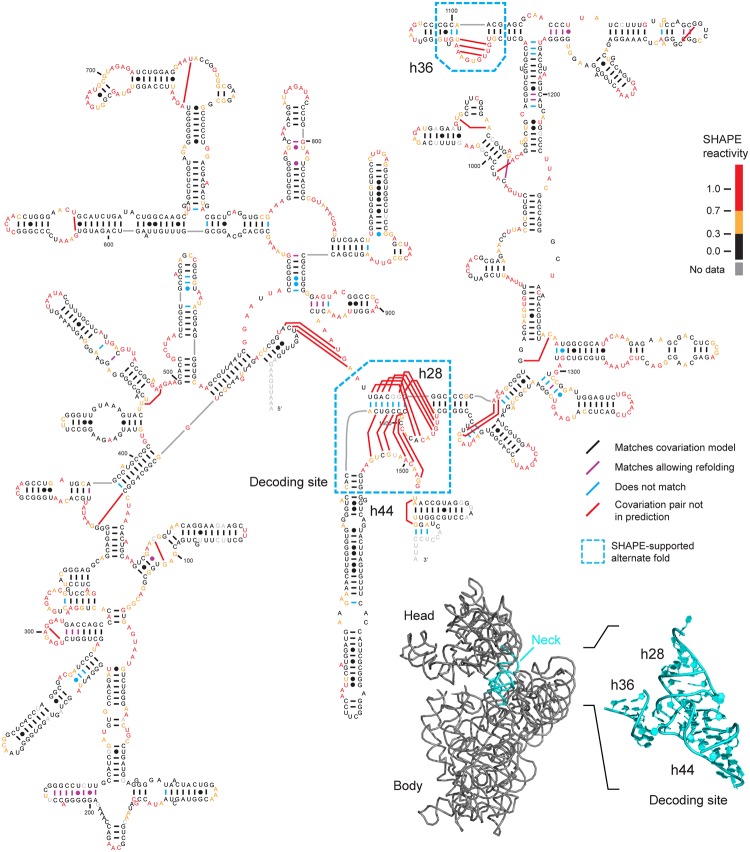
Secondary structure model for *E*. *coli* 16S rRNA. This model was constrained by 16S rRNA consensus base pairs derived from SHAPE-based sequence alignment. Predicted pairs that exactly match the accepted covariation model [11] are shown with short black lines, and predicted pairs that match after modest local refolding are purple. Predicted pairs not in the covariation model are illustrated with blue lines. Covariation pairs not in the SHAPE-aligned structure are shown using red lines. *E*. *coli* SHAPE reactivities are shown by coloring of individual nucleotides (see scale). Areas with large-scale SHAPE-supported alternative folds are emphasized with cyan boxes. These areas (cyan) are illustrated on a structure model of the 16S ribosome [38] (bottom right) and cluster in the neck and decoding site. The inset is shown with an orientation that allows both h36 and the decoding site (h28 and h44) to be seen clearly.

Structure models were generated for 16S and 23S ribosomal RNAs (Fig 3; S1 and S2 Figs). The modeled structures were compared to those based on covariation models in the CRW. A local refolding allowance of 5 nucleotides was used in sensitivity and positive predictive value calculations (Methods) to allow for modest local rearrangements of base pairs, which we find are broadly supported by experimental SHAPE reactivities (Fig 3; S1 and S2 Figs). All SHAPE reactivity-based structures had sensitivities greater than 90% relative to the accepted structures, indicating that models were of high accuracy (Table 2, column 5). Given the strong dependence of covariation-based prediction approaches on alignment quality, this successful modeling further validated the strong utility of SHAPE-structure alignment of RNA sequences. Importantly, a significant subset of base pairs in the reference covariation models were incompatible with observed SHAPE reactivities. Of base pairs in reference covariation models not supported by SHAPE data, a significant number involve one or more nucleotides with SHAPE reactivities greater than or equal to 0.5 (*E*. *coli* 16S, 70%; *C*. *difficile* 16S, 64%; *H*. *volcanii* 16S, 39%; *E*. *coli* 23S, 46%). This suggests that these nucleotides are weakly base paired or form alternate structures under the experimental conditions used in this work.

Secondary structure predictions based on SHAPE-directed alignment were compared with sequence-only predictions and with predictions directed by sequence-only alignment or SHAPE reactivities (Table 2). Secondary structure predictions for ribosomal RNA based on sequence alone have sensitivity values of roughly 65% (predictions by RNAfold of the Vienna RNA package). At this accuracy, a bare majority of base pairs are predicted correctly, and it remains difficult to develop meaningful biological hypotheses regarding RNA structure. When we used sequence-only alignments to constrain secondary structure prediction using RNAalifold [21], *E*. *coli* and *C*. *difficile* 16S rRNA predictions improved (Table 2), but *H*. *volcanii* 16S and *E*. *coli* 23S rRNA predictions actually had lower sensitivities. The mixed results of structure modeling based on sequence-only alignments reflect the sensitivity of a prediction to the accuracy of the initial alignment. Secondary structure predictions directed by SHAPE reactivities improve sensitivity relative to predictions based only on thermodynamic parameters [4, 23], and models that consider sequence alignment and SHAPE reactivities had higher sensitivity and positive predictive values than models considering SHAPE data alone (Table 2).

## Discussion

### Applications of SHAPE-based comparisons

Functional RNA elements commonly exhibit conservation at the levels of both sequence and structure, and wide variations in primary sequence can be compatible with nearly identical higher-order structures [2, 3]. Thus, incorporating a model-free metric for RNA structure, such as SHAPE reactivity, holds significant promise in functional RNA motif discovery. In the case of the ribosomal RNAs, sequence alignments based on SHAPE reactivities were roughly as accurate as approaches that used only sequence information (Table 1). When both SHAPE reactivities and base identity were considered together, alignment quality increased. Alignments taking into account both SHAPE reactivities and base identity achieved sensitivities exceeding 90% for alignments of *E*. *coli* ribosomal RNA to *C*. *difficile* sequences.

SHAPE reactivities provide information that is orthogonal to the sequence of nucleobase identities [24]. SHAPE-based alignments were performed with a dynamic programming algorithm using a pair-wise scoring system, analogous to the substitution matrices commonly used in standard alignment approaches. Given this, the scoring system should be broadly applicable to other sequence alignment approaches, including methods that use heuristic scoring systems, such as BLAST [25], or probabilistic approaches, such as trained Markov-based alignment methods [26]. Given the success using datasets generated efficiently by massively parallel sequencing using the MaP approach [10], SHAPE-based alignments will likely prove broadly useful in future high-throughput structure-based RNA motif discovery and structure modeling.

### Conserved, alternate base-pairing conformations in 16S rRNA

**Fig 4:**
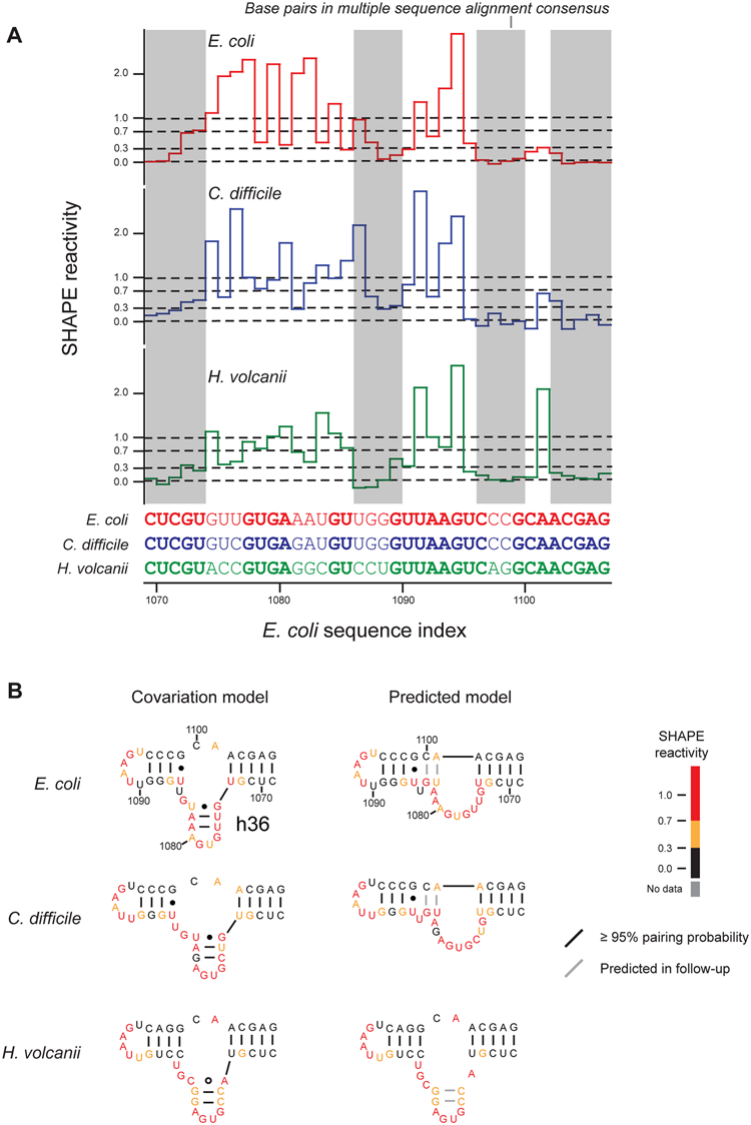
Consensus alternate structures for helix 36 of *E*. *coli* 16S rRNA. (**A**) SHAPE reactivities for aligned regions with consensus areas (RNAalifold) highlighted in gray. (**B**) Structures for the covariation and SHAPE-structure constrained models. Base pairs predicted in the first-step (RNAalifold) consensus are black, and base pairs predicted in the follow-up constrained (RNAfold) prediction are shown in gray.

**Fig 5:**
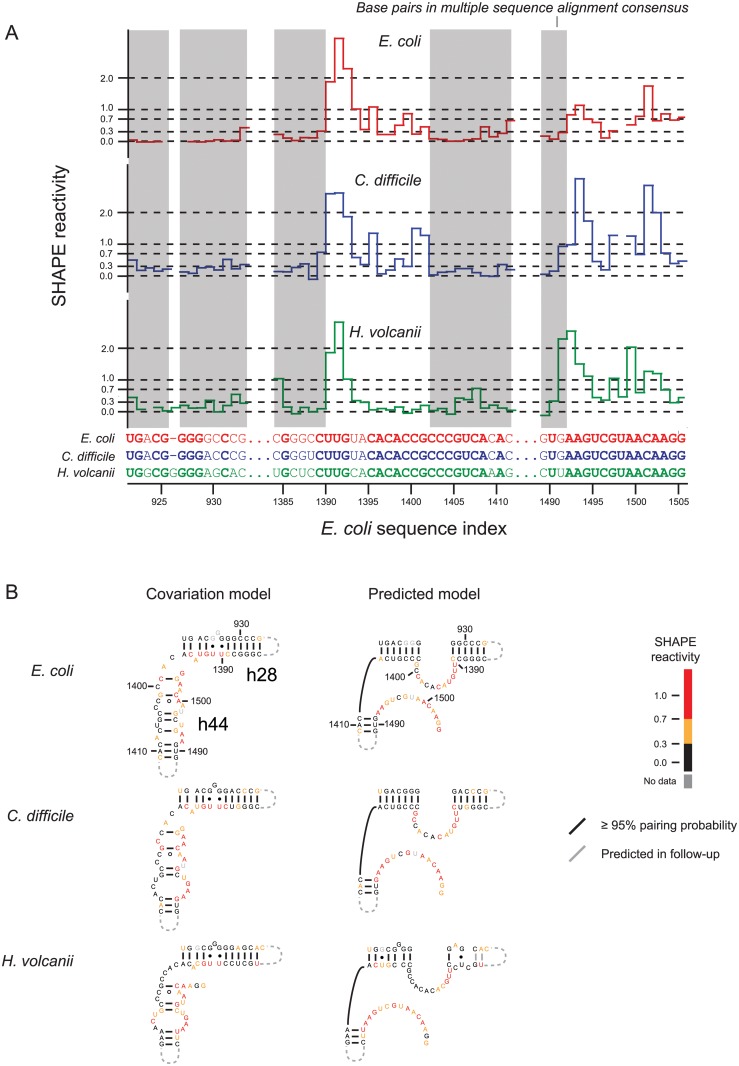
Consensus alternate structures in the decoding site. Helices 28 and 44 of the E. coli 16S rRNA are shown. (**A**) SHAPE reactivities for aligned regions. Consensus base pairs are highlighted in gray. (**B**) Structures for the covariation and SHAPE-structure constrained models. Base pairs in the RNAalifold consensus are shown in black, and base pairs predicted in the follow-up constrained RNAfold prediction are gray.

Secondary structure models, generated from SHAPE-based alignment, include the vast majority of base pairs in the accepted covariation-based models of the 16S and 23S ribosomal RNAs (Fig 3 and Tables 1 and 2). However, there was notable localized disagreement between the alignment-based predictions from this work and covariation models in two regions in the 3' major domain of 16S rRNA: in helix 36 (h36; Figs 3 and 4) and in the decoding site (h28 and h44; Figs 3 and 5). These alternate structures are predicted to exist in each of the *E*. *coli*, *C*. *difficile*, and *H*. *volcanii* 16S rRNAs. Moreover, in each case, the alignment-directed model shows much better agreement with the experimental SHAPE reactivities than does the covariation model. For example, positions corresponding to base pairs in h36 in the covariation structure had high SHAPE reactivities (Fig 4B, in red), indicating structural flexibility. Similarly, many nucleotides in the h28 and h44 helices in the covariation model were highly reactive to the SHAPE reagent and were not present in the alignment-based consensus model (Fig 5B). For the h28 and h44 helices, the SHAPE-based alignment and the underlying individual nucleotide reactivities strongly support a novel alternate base-paired secondary structure (Fig 5). Taken together, the SHAPE reactivity data indicate that these alternate structures describe the conformation predominantly assumed by the protein-free rRNA sampled during chemical probing. Exclusion of these regions from the *E*. *coli* 16S rRNA sensitivity calculations increased the sensitivity of the secondary structure prediction from 93.8% to 96.8% (Table 2, column 6).

Ribosomal RNAs were assayed in the absence of protein to generate the SHAPE data used in this study. Total cellular RNA was obtained from each organism under conditions that avoided denaturing or heating steps and were thus supportive of maintenance of native-like RNA structure. We therefore infer that the alternate 16S secondary structure discovered in this work describes a low-energy state that is readily sampled by protein-free ribosomal RNA. Crystallographic studies of the ribosomal subunits are in agreement with the structure inferred by covariation analysis. However, given that the alternate conformation predominates for the free RNA based on SHAPE analysis, this alternate state may be adopted by ribosomal RNA prior to small subunit assembly and may reflect a conformation that occurs during small subunit assembly or during specific phases of the translation cycle. This view is supported by nucleotide-resolution chemical modification and interference assays that suggest that nucleotides in h36 and in the decoding site (h28 and h44) undergo a conformational change during conversion of the 30S subunit from an inactive to active conformation [27] and are involved in rate determining steps during 30S subunit assembly [28, 29]. In addition, helices in the decoding region (h28 and h44) are conspicuously lacking base-pair level covariation [30]. The striking absence of covariation support is consistent with the idea that these helices are subject to additional structural constraints beyond formation of a single set of conventional helices and instead form additional conserved alternate secondary structures. Critically, no covariation or other evidence contradicts the alternate base pairs proposed here.

Strikingly, all regions that structure-directed alignment suggests form alternate structures in the 16S rRNA are located close to each other in three-dimensional space, in the neck of the intact small ribosomal subunit (Fig 3, *inset*). Given that these alternate structures occur in and near the codon-anticodon decoding site, we propose that these structures form a switch involving low energy base-pairing rearrangements important for regulation of translation.

### Perspective

The strategies developed here allow for highly accurate, structure-informed alignments of large (Table 2) and likely small (Fig 2) RNAs and are accurate even for RNAs that show high levels of sequence divergence. These alignments may in turn be used to predict consensus secondary structures with high accuracy. Given that structural information is derived from the SHAPE-MaP high-throughput strategy for nucleotide-level quantitation of chemical probing data [10], this approach for structure-informed alignment and secondary structure modeling will be broadly useful for large-scale analysis of entire transcriptomes and large families of functional RNA molecules. Using SHAPE-structure informed alignments, we discovered structural rearrangements in the base pairing patterns of 16S that are conserved in three diverse organisms. These structural rearrangements are likely to have mechanistic functions in ribosome assembly or regulation of translation. This analysis of ribosomal RNA indicates that SHAPE-based alignment methods will prove especially powerful in discovering functional motifs in RNA elements with low sequence covariation.

## Methods

### *E*. *coli* ribosomal RNA SHAPE-MaP data

Total *E*. *coli* RNA (DH5*α* strain) was prepared as described [4]; SHAPE-MaP data for the ribosomal RNAs were reported previously [10].

### Preparation of *C*. *difficile* ribosomal RNA

*C*. *difficile* (strain 630) was grown in BHIS medium [31] at 37°C under anaerobic conditions (90% N_2_, 5% CO_2_, and 5% H_2_) [32] to an OD_600_ of 1.0. Cells were collected by centrifugation (10 min, 4°C, 4000×g). The pellet was washed with 1× TE [10 mM Tris (pH 8.0), 1 mM EDTA]. The supernatant was discarded, and the pellet was allowed to air-dry for 5 minutes. To lyse the cells, 1 mL TRIsure (Bioline) was added to the pellet. The resultant mixture was incubated at room temperature for 5 minutes. The mixture was then transferred to a vial containing 250 μL 0.1-mm glass beads. Cells were lysed using a bead beater over two 90 second pulses, with cells held on ice between pulses. The resultant mixture was extracted with 200 μL chloroform, and the aqueous layer was extracted three times with phenol (pH 8.0):chloroform:isoamyl alcohol (25:24:1), followed by three extractions with chloroform. The RNA-containing solution was exchanged for folding buffer (50 mM Hepes (pH 8.0), 200 mM potassium acetate (pH 8.0), and 5 mM MgCl_2_) using a pre-equilibrated gel filtration column (G-25 column, GE).

### Preparation of *H*. *volcanii* ribosomal RNA

Growth medium was prepared by bringing 600 ml 30% salt solution [4 M sodium chloride, 150 mM magnesium chloride hexahydrate, 150 mM magnesium sulfate heptahydrate, 100 mM potassium chloride, 5 mM Tris (pH 7.5)], 5 g bacteriological peptone (LP37; Oxoid), and 1 g yeast extract (LP21; Oxoid) to 1 L with deionized water. *H*. *volcanii* cells (strain DS70) were grown to an OD_600_ of 0.8 and collected by centrifugation (5 min, 4°C, 14000×g). Cells were lysed by incubation in low salt solution [220 μL 50 mM Hepes (pH 8.0) and 5 mM MgCl_2_; incubation at 22°C for 5 min, followed by incubation on ice for 5 min]. Following lysis, this solution was extracted three times with phenol (pH 8.0):chloroform:isoamyl alcohol (25:24:1), followed by three extractions with chloroform. The RNA-containing solution was exchanged for folding buffer [50 mM Hepes (pH 8.0), 200 mM potassium acetate (pH 8.0), and 5 mM MgCl_2_] using a pre-equilibrated gel filtration column (G-25 column, GE).

### SHAPE-MaP characterization of ribosomal RNA

Determination of SHAPE reactivity by SHAPE-MaP employs three experiment conditions: chemical modification of native RNA, chemical modification of denatured RNA, and a no-modification control. All chemical modifications were performed using 1-methyl-7-nitroisatoic anhydride (1M7) [33]. Chemical modification of native RNA and the no-modification control were performed in parallel. To 1× folding buffer [50 mM HEPES (pH 8.0), 200 mM potassium acetate (pH 8.0), and 5 mM MgCl_2_] was added to a concentrated RNA solution (280 ng *H*. *volcanii* total RNA or 70 ng *C*. *difficile* total RNA; amounts determined using absorption spectroscopy) to a final volume of 90 μL. The RNA solution was incubated at 37°C for 30 minutes. Following incubation, 10 μL DMSO (no-modification control) or 10 μL 100 mM 1M7 in DMSO (native 1M7-modified sample) was added to the RNA solution. The RNA solution was then incubated at 37°C for 3 minutes. For the denatured control, 25 μL 4× denaturing control buffer [200 mM HEPES (pH 8.0), 16 mM EDTA] and 50 μL deionized formamide were added to a concentrated RNA solution (280 ng *H*. *volcanii* or 70 ng *C*. *difficile* total RNA), and deionized water was added to a final volume of 90 μL. This solution was held at 95°C for 1 minute, and then 10 μL 100 mM 1M7 in DMSO was added; the combined solution was incubated at 95°C for 1 minute.

### Library preparation for sequencing

After modification, all three samples were purified by affinity chromatography (RNeasy Min-Elute; Qiagen) with elution into 22 μL buffer. To prepare sequencing libraries, the purified RNA samples were first fragmented; 20 μL of RNA solution was combined with 30 μL fragmentation buffer [250 mM Tris (pH 8.3), 375 mM KCl, 15 mM MgCl_2_], incubated at 94°C for 4 minutes, and then transferred immediately to ice. Fragmented RNA was purified using a G-25 column (GE) with elution into 1× TE [10 mM Tris (pH 8.0), 1 mM EDTA]. Following fragmentation, reverse transcription was performed using a 20-μL aliquot of fragmented RNA and 2 μL random DNA nonamers (200 ng/μL). The solution was incubated at 65°C for 5 minutes and then placed on ice. To this solution, 7 μL reaction buffer [286 mM Tris (pH 8.0), 429 mM KCl, 57 mM DTT, 2.9 mM dNTP mix (dATP, dCTP, dGTP, and dTTP, 2.9 mM each)], 4 μL 60 mM MnCl_2_, and 5 μL water were added. The solution was pre-incubated at 25°C for 2 minutes prior to adding 2 μL Superscript II (Invitrogen). The reaction was incubated at 25°C for 10 minutes, 42°C for 180 minutes, and 70°C for 15 minutes. Following reverse transcription, the RNA was purified using a G-25 column (GE) with elution into 1× TE.

The cDNA was converted to a double-stranded DNA library with Illumina platform-specific sequence tags. First, 40 μL of the purified reverse transcription product was used in an 80-μL second-strand synthesis reaction (NEBNext Second Strand Synthesis Module, New England Biolabs). The product of the second-strand synthesis reaction was purified (PureLink PCR Micro Kit; Life Technologies) and eluted into 12 μL elution buffer. A 10-μL aliquot of the purified DNA solution was then used in a 50-μL end repair reaction (NEBNext End Repair Module, New England Biolabs). Following end repair, the DNA was purified (1.6× Ampure XP Bead clean-up; Agencourt, Beckman Coutler) and eluted into a final volume of 30 μL 1× TE.

To incorporate Illumina platform-specific sequence tags, a dA-tailing reaction was used to incorporate a single-nucleotide overhang at the 3′ ends of the double-stranded DNA. A 15-μL aliquot of purified DNA from the end repair step was used in a 20-μL dA-tailing reaction (NEBNext dA-Tailing Module, New England Biolabs). Illumina sequences were incorporated using a ligation step with Illumina iAdapters (prepared in house). Immediately following completion of the dA-tailing reaction, 7.5 μL of 5× reaction buffer (NEBNext Quick Ligation Module, New England Biolabs), 2.5 μL 125 nM DNA adapter, 3.75 μL Quick T4 DNA Ligase (New England Biolabs), and 3.75 μL water were added to the dA-tailing reaction mix. The ligation reaction was then incubated at 20°C for 15 minutes. The ligation reaction was purified twice (1.0× Ampure XP Bead clean-up; Agencourt, Beckman Coutler) with final elution into 20 μL 10 mM Tris (pH 8.0).

Illumina libraries were prepared using emulsion PCR [10, 34]. The aqueous phase was composed of 5 μL of double-stranded DNA, 10 μL 10 μM Illumina-specific forward strand primer, 10 μL 10 μM Illumina-specific reverse strand primer, 40 μL Q5 5× reaction buffer (New England Biolabs), 100 μL 20 g/L bovine serum albumin, 4 μL dNTP mix (10 mM each, dATP, dCTP, dGTP, dTTP), 2 μL Q5 high-fidelity polymerase (New England Biolabs), and 29 μL water. The DNA was amplified in a 35-cycle PCR reaction (denaturation: 94°C for 30 sec; annealing: 67°C for 30 sec; extension: 72°C for 30 sec). To purify the PCR product, the reaction was first applied to a PureLink PCR cleanup column (Life Technologies). The column eluent was then purified using a 1.0× Ampure XP Bead clean-up (Agencourt, Beckman Coutler). This bead cleanup was performed twice with elution into 12 μL 10 mM Tris (pH 8.0).

The concentrations of sequencing samples were determined by Qubit High Sensitivity DNA fluorescence assays (Life Technologies) and High Sensitivity DNA Bioanalyzer assays (Agilent). Each sample was diluted to 2 nM and pooled. The pooled library was sequenced using an Illumina MiSeq (300 cycles—PE kit). Sequences were aligned and mutation events counted using the SHAPE-MaP analysis pipeline [10]. SHAPE reactivities were computed based on mutation rates in the native 1M7-modified sample, minus the denatured 1M7-modified sample, and normalized by the background control.

### SHAPE-based RNA sequence alignment

SHAPE-based alignment was based on the Gotoh algorithm with affine gap penalties [35]. SHAPE-based alignment of two sequences *x* and *y* began with declaration of matrices *D*, *P*, and *Q*. Each matrix had dimensions *m* by *n*, where *m* and *n* were the lengths of sequences *x* and *y* plus 1, respectively. Considering alignment of (*x*_*0*_…*x*_*i*_) and (*y*_*0*_…*y*_*j*_), *D*_*i*,*j*_ corresponds to the score associated with alignment, *P*_*i*,*j*_ corresponds to the score associated with alignment that ends with a gap in *x*, and *Q*_*i*,*j*_ corresponds to the score of alignment that ends with a gap in *y*. To initialize each matrix, *D*_*0*,*0*_ was set to 0, *D*_*i*,*0*_ was set to *GOP* + *i* × *GEP*, and *D*_*0*,*j*_ was set to *GOP* + *j* × *GEP*, where *GOP* and *GEP* are the gap opening penalty and gap extension penalty, respectively; *P*_*0*,*j*_ and *Q*_*i*,*0*_ were set to arbitrarily large negative numbers. Every other cell in the matrix was populated by the following recursion, where *s*(*x*_*i*_, *y*_*j*_) describes a pair-wise comparison score, and *x*_*i*_ and *y*_*j*_ are the SHAPE values of each sequence at *i*-th and *j*-th positions:

Pi,j = maxPi-1,j+GEPDi-1,j+GOP+GEP

Qi,j = maxQi,j-1+GEPDi,j-1+GOP+GEP

Di,j = maxsxi,yj+Di-1,j-1Pi,jQi,j

The scoring function (see Fig 1B) is described by the following equation, with parameters *m* and *b*:

sxi,yj = maxmxi-yj+b-m+b

If base identity was taken into account during alignment, it was added as an additional scoring term *b* in the recursion, where *x'*_*i*_ and *y'*_*j*_ were the base identities at positions *i* and *j* in sequences *x* and *y*, respectively:

Pi,j = maxPi-1,j+GEPDi-1,j+GOP+GEP

Qi,j = maxQi,j-1+GEPDi,j-1+GOP+GEP

Di,j = maxsxi,yj+bx'i,y'j+ Di-1,j-1Pi,jQi,j

The scoring function *b* is described by the following equation with parameters *MATCH* and *MISMATCH*.

bx'i,y'j = MATCH,x'i = y'jMISMATCH,x'i≠y'j

Following population of the matrices by recursion, a trace-back operation was used to find the optimal alignment. The trace-back operation began at position *i*,*j*, representing the 3′-most position of the alignment. The next position in the alignment was found using the following comparison: if *D*_*i*,*j*_ was equal to sum of *D*_*i-1*,*j-1*_ and the score of the pairwise comparison between *x*_*i-1*_ and *y*_*j-1*_, the next position was aligned nucleotides *x*_*i-1*_ and *y*_*i-1*_; if *D*_*i*,*j*_ was equal to *P*_*i*,*j*_, the next position was a gap in sequence *y*; if *D*_*i*,*j*_ was equal to *Q*_*i*,*j*_, the next position was a gap in sequence *x*. The trace-back operation was finished when a position was encountered where *i* = 0 or *j* = 0.

The SHAPE scoring parameters *m* and *b*, base-identity scoring parameters *MATCH* and *MISMATCH*, and gap penalty parameters *GOP* and *GEP* were optimized by exhaustive search over the 16S and 23S rRNA aligned sequence pairs. The best parameter set was then selected based on the average sensitivity across all alignments. In experiments considering only SHAPE values, *m* = -2, *b* = 2, *GOP* = -5, and *GEP* = -0.25. Scoring function parameters were preserved across SHAPE-only and combined SHAPE and base identity alignments, but *GOP* and *GEP* parameters were reoptimized for alignments considering both SHAPE reactivity and base identity. When both SHAPE and base identity were considered, *m* = -2, *b* = 2, *GOP* = -6, *GEP* = -1, *MATCH* = 2, and *MISMATCH* = -2.

### Evaluation of RNA sequence alignments

From multiple sequence alignments on the CRW, pairwise alignments between *E*. *coli* and *C*. *difficile* and *E*. *coli* and *H*. *volcanii* were obtained for both 16S and 23S rRNAs. RNA sequence alignments generated in this work were then evaluated by comparison to these alignments. Sensitivities were calculated as the percentage of matched nucleotides in the CRW alignments found in a given alignment.

### Multiple sequence alignments

Multiple sequence alignments were generated using T-Coffee [16]. First, pairwise alignments were generated for all possible pairs between sequences under consideration. These pair-wise alignments were then used as arguments for T-Coffee using default parameters. T-Coffee creates a multiple sequence alignment based on the consensus of individual alignments. Only alignments generated by the methods described in this work were used to make multiple sequence alignments.

### Secondary structure modeling by SHAPE-based RNA alignments

To establish a base-line for comparison, sequence-only secondary structure predictions were performed with RNAfold from the Vienna RNA package with a maximum base paring distance of 600 nucleotides [20]. Secondary structure models were also generated using sequence-only alignments or SHAPE reactivities. Pairwise Needle alignments were used to generate a multiple sequence alignment using T-Coffee [16]. This multiple sequence alignment was in turn used by RNAalifold [21] to create a consensus secondary structure. Default RNAalifold parameters were used with the exceptions that the ribosum matrix [36] and a maximum base pairing distance of 600 nucleotides were imposed. Individual SHAPE-directed predictions were made using RNAfold, with SHAPE data incorporated as a pseudo-free energy term [4, 23].

Multiple sequence alignments were used as input for RNAalifold from the Vienna RNA package [21]. SHAPE data were incorporated as an additional pseudo-free energy term to constrain secondary structure prediction [4, 23] using a new implementation of the RNAalifold algorithm. Secondary structure prediction and partition function calculations were performed using the ribosum matrix [36] with a maximum base pairing distance of 600 nucleotides. Following RNAalifold modeling, all base pairs in the consensus sequence with pairing probabilities greater than 95% were used as constraints in individual follow-up models using RNAfold, also of the Vienna RNA package [20]. SHAPE data were also used to constrain secondary structure modeling in this step, using a maximum base pairing distance of 600 nucleotides. The SHAPE-aware implementations of RNAfold and RNAalifold are part of the upcoming release of the Vienna RNA Package 2.2. A release candidate of this software is available at http://www.tbi.univie.ac.at/RNA.

### Evaluation of secondary structure models

Secondary structure models were evaluated by calculating sensitivity (sens) as the percentage of base pairs from the CRW covariation model found in predicted structures and by calculating positive predictive values (ppv) as the percentage of predicted pairs found in the covariation model. It should be emphasized that these reference structures are themselves experimental models, and base pairs in these models may show slight local rearrangements in terms of base-pairing partners [4, 37]. To account for this, when comparing base pairs between the covariation and predicted models, a modest local refolding allowance of 5 nucleotides was permitted. To be considered matched, a base pair in the covariation model at positions *x* and *y* and any base pair in the predicted model at positions *xʹ* and *yʹ* were required to meet the following criterion:

$$\left[x\;=\;x'and\;\left|y-y'\right|\leq 5]\;or\;[y\;=\;y'and\;\left|x-x'\right|\leq 5\right]$$

Pseudoknots and non-canonical base pairs (with the exception of G-U pairs) were not considered in sens and ppv calculations.

## Supporting Information

S1 Fig Secondary structure model for the *E*. *coli* 23S rRNA, nucleotides 1–1646. This model was constrained by 23S rRNA consensus base pairs based on a SHAPE-based sequence alignment. Predicted pairs that exactly match the accepted covariation model [11] are shown in black, and predicted pairs that match after allowing modest local refolding are purple. Predicted pairs not in the covariation model are blue. Covariation pairs not in the SHAPE-aligned structure are shown using red lines. Individual *E*. *coli* nucleotides are colored by their SHAPE reactivities (see scale).
(TIF)

S2 Fig Secondary structure model for *E*. *coli* 23S rRNA, nucleotides 1647–2904. Full legend is given in S1 Fig.
(TIF)

S1 Dataset Complete SHAPE and alignment datasets for the 16S and 23S rRNAs from *E*. *coli*, *C*. *difficile* and *H*. *volcanii*. (ZIP)

S1 Software Code for the alignment and secondary structure modeling algorithms developed in this work. (ZIP)
