# Supplementary figures and images for: Model-Free RNA Sequence and Structure Alignment Informed by SHAPE Probing Reveals a Conserved Alternate Secondary Structure for 16S rRNA
Source: PLoS Comput Biol. 2015 May 20;11(5):e1004126. doi: 10.1371/journal.pcbi.1004126 (PMC4438973; doi:10.1371/journal.pcbi.1004126)

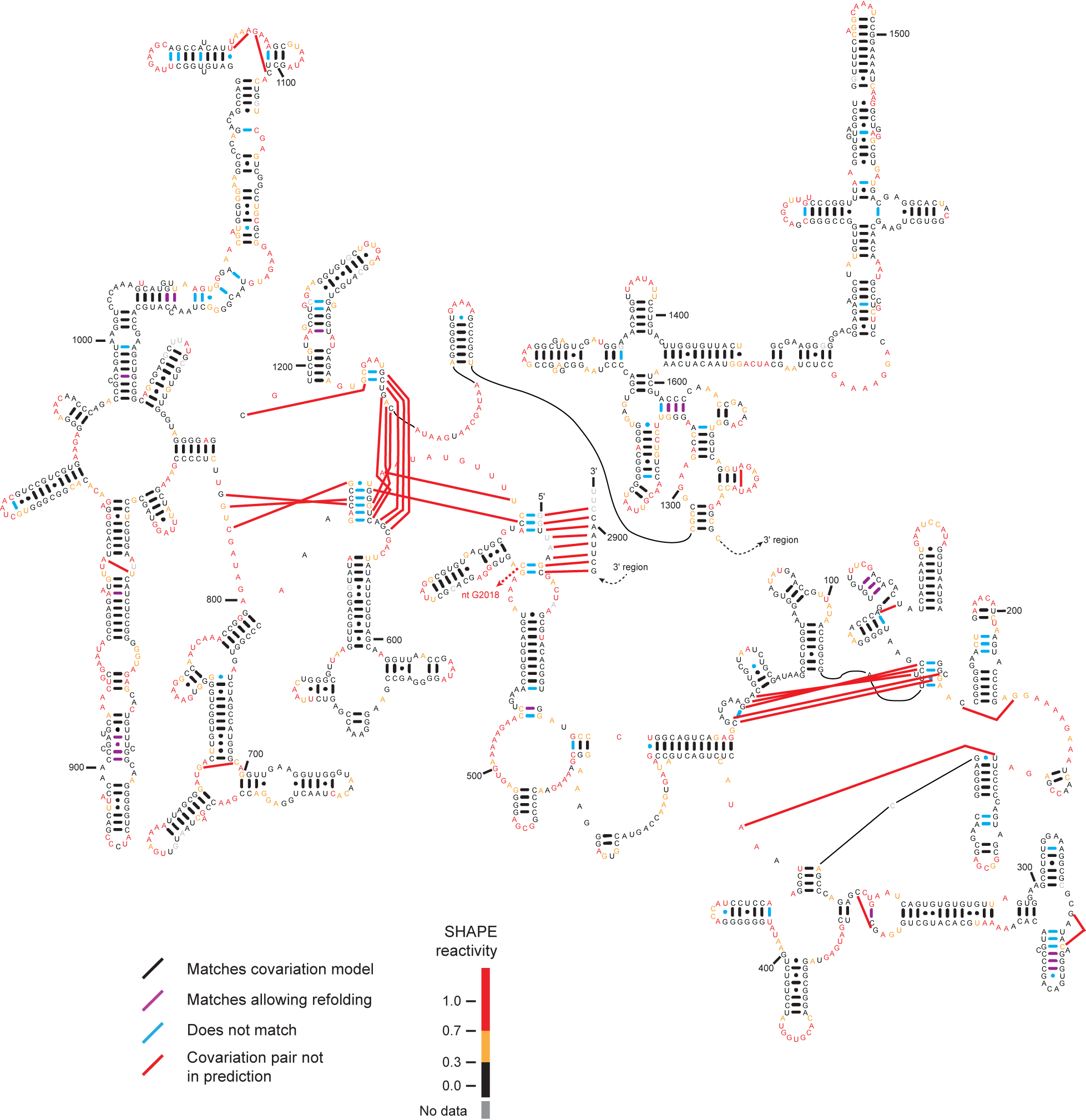

Supplement: S1 Fig — This model was constrained by 23S rRNA consensus base pairs based on a SHAPE-based sequence alignment. Predicted pairs that exactly match the accepted covariation model [11] are shown in black, and predicted pairs that match after allowing modest local refolding are purple. Predicted pairs not in the covariation model are blue. Covariation pairs not in the SHAPE-aligned structure are shown using red lines. Individual E. coli nucleotides are colored by their SHAPE reactivities (see scale). (TIF) [file pcbi.1004126.s001.tif]

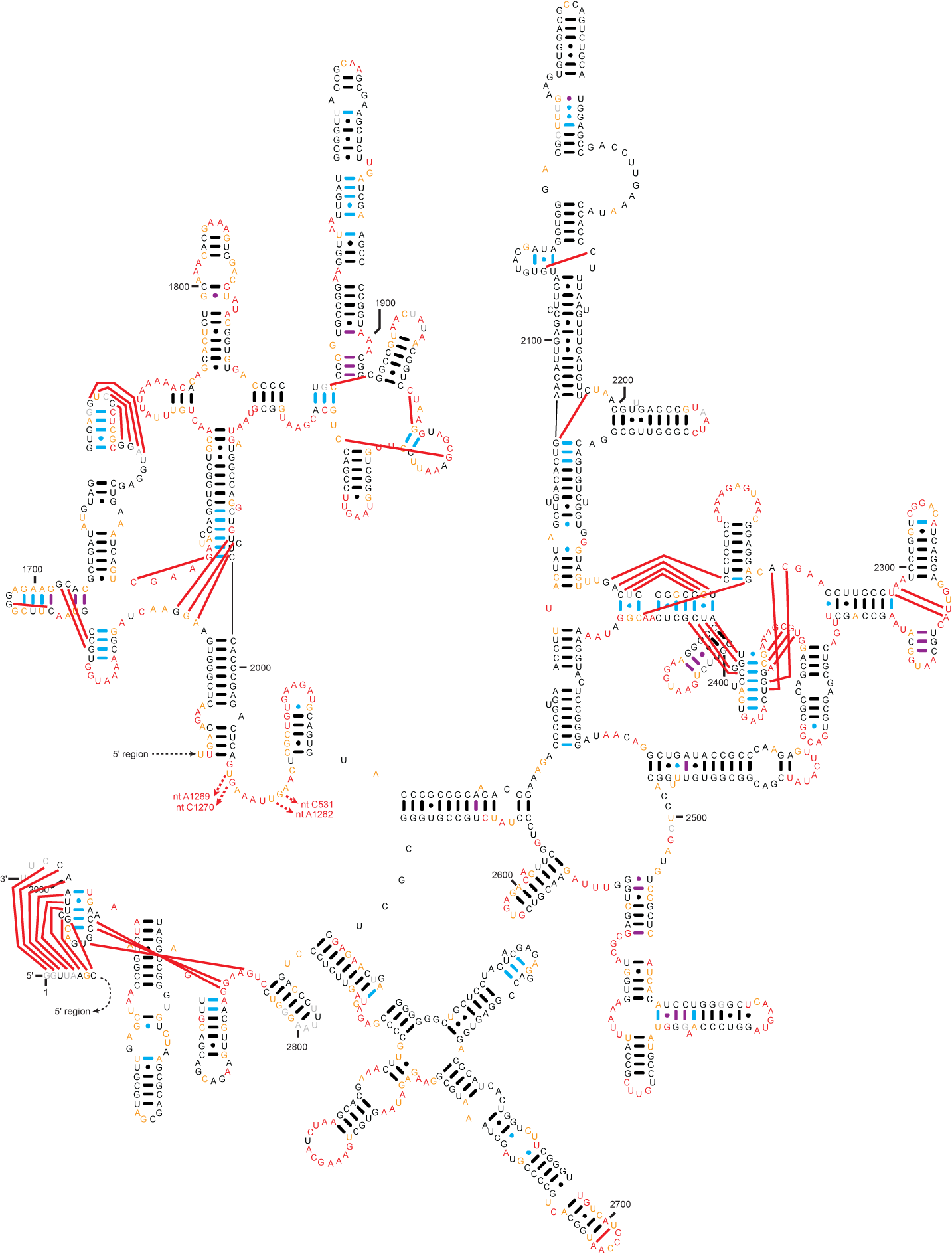

Supplement: S2 Fig — Full legend is given in S1 Fig. (TIF) [file pcbi.1004126.s002.tif]
